# Aromatase Expression Increases the Survival and Malignancy of Estrogen Receptor Positive Breast Cancer Cells

**DOI:** 10.1371/journal.pone.0121136

**Published:** 2015-04-02

**Authors:** Keya De Mukhopadhyay, Zhao Liu, Abhik Bandyopadhyay, Nameer B. Kirma, Rajeshwar R. Tekmal, Shui Wang, Lu-Zhe Sun

**Affiliations:** 1 Department of Cellular and Structural Biology, University of Texas Health Science Center, San Antonio, United States of America; 2 Department of Obstetrics and Gynecology, University of Texas Health Science Center, San Antonio, United States of America; 3 Department of Molecular Medicine, University of Texas Health Science Center, San Antonio, United States of America; 4 Cancer Therapy and Research Center, University of Texas Health Science Center, San Antonio, United States of America; 5 Department of Breast Surgery, the First Affiliated Hospital of Nanjing Medical University, Nanjing, China; University of South Alabama, UNITED STATES

## Abstract

In postmenopausal women, local estrogen produced by adipose stromal cells in the breast is believed to support estrogen receptor alpha (ERα) positive breast cancer cell survival and growth. This raises the question of how the ERα positive metastatic breast cancer cells survive after they enter blood and lymph circulation, where estrogen level is very low in postmenopausal women. In this study, we show that the aromatase expression increased when ERα positive breast cancer cells were cultured in suspension. Furthermore, treatment with the aromatase substrate, testosterone, inhibited suspension culture-induced apoptosis whereas an aromatase inhibitor attenuated the effect of testosterone suggesting that suspended circulating ERα positive breast cancer cells may up-regulate intracrine estrogen activity for survival. Consistent with this notion, a moderate level of ectopic aromatase expression rendered a non-tumorigenic ERα positive breast cancer cell line not only tumorigenic but also metastatic in female nude mice without exogenous estrogen supplementation. The increased malignant phenotype was confirmed to be due to aromatase expression as the growth of orthotopic tumors regressed with systemic administration of an aromatase inhibitor. Thus, our study provides experimental evidence that aromatase plays an important role in the survival of metastatic ERα breast cancer cells by suppressing anoikis.

## Introduction

Breast cancer is the most frequent malignant disease in women that affects 1 in 8 North American women throughout their lifetime and is the second leading cause of cancer-deaths in the U.S.[1]. Successful treatment of malignant breast cancer remains a challenge to the medical professionals due to the frequent failure of chemotherapy, endocrine therapy or immunotherapy.

Excessive estrogen exposure is a well-recognized critical risk factor for breast cancer. Ovaries are the principal source of systemic estrogen in the premenopausal women[2]. Other sites of estrogen biosynthesis which become the major sources after menopause includes mesenchymal cells of the adipose tissue and skin, osteoblasts and chondrocytes in bone, vascular endothelial and aortic smooth muscle cells along with a number of sites in the brain including medial preoptic/anterior hypothalamus, the medial basal hypothalamus, and the amygdale. The estrogen synthesized within these extragonadal sites is probably only biologically active at a local tissue level in a paracrine or intracrine fashion[3, 4]. The total amount of estrogen synthesized by these extragonadal sites may be small, but the local tissue concentrations achieved are probably high and exert significant biological influence locally[3]. After menopause, the mesenchymal cells of the adipose tissue become the main source of estrogen. Therefore, in the post-reproductive years, the degree of a woman's estrogenization is mainly determined by the extent of her adiposity: corpulent women are protected against osteoporosis; conversely obesity is positively correlated with breast cancer risk[5, 6]. In postmenopausal women the level of circulating estrogens are greatly diminished. Yet, their breast cancer is mostly estrogen receptor positive (ER+) and relies heavily on the estrogens for the survival and progression of the disease. These estrogens are synthesized in the extragonadal sites such as adipose tissue, bone and brain because they express aromatase (Aro, *Cyp19*), which is a key enzyme in estrogen biosynthesis and an important target in breast cancer therapy.

Aromatase P450 (*CYP19*) converts testosterone to estradiol, which is the final and rate-limiting step in estrogen biosynthesis. Inhibition of aromatase effectively eliminates estrogen production in the entire body[3, 7, 8]. As aromatase abundance is a critical determinant for local and circulating estrogen levels, regulation of tissue-specific aromatase expression has a prominent impact on different estrogen target tissues under physiological as well as pathological conditions. Aromatase inhibitors (AIs) such as letrozole inhibit the enzymatic activity of aromatase and thereby diminish the estrogenic capability throughout the body. They are widely used as the first line therapy in postmenopausal women with ER+ breast cancer[9, 10]. In fact, AIs are proving to be more effective than tamoxifen for postmenopausal patients with breast cancer[9].

Although the estrogens produced by breast adipose tissue in postmenopausal women are strongly implicated in promoting the development and growth of ER+ breast cancer in the breast, it is unclear how metastatic ER+ breast cancer cells stay viable in circulation after intravasation and leaving the estrogen-rich mammary microenvironment. In the current study, we show that aromatase expression is increased when ER+ breast cancer cells were suspended in culture media. Interestingly, while suspension culture caused significant apoptosis called anoikis, addition of testosterone completely blocked anoikis in the absence of an AI, but not in the presence of the AI. Significantly, ectopic expression of aromatase made a non-tumorigenic ER+ breast cancer cell line not only tumorigenic and but also metastatic in the absence of exogenous supplementation of estrogen in female athymic nude mice.

## Materials and Methods

### Ethics statement

All animal protocols were approved by the Institutional Animal Care and Use Committee of the University of Texas Health Science Center at San Antonio. All animal experiments were monitored by the Department of Laboratory Animal Resources at the University of Texas Health Science Center at San Antonio.

### Cell lines and culture

The human breast cancer cell line ZR-75-1 was originally obtained from the American Type Culture Collection (Manassas, VA). We stably transfected the ER positive breast cancer ZR-75-1 cells with an human aromatase expression vector and obtained a few clones expressing a moderately higher level of aromatase in comparison with the control vector-transfected cells. One of the clones called ZR-75-1/Aro-Clone10 (Cl.10) was selected for further study due to its relatively higher aromatase level than other clones. Cl.10 cells formed tumors after being xenografted into the inguinal mammary fat pad of the female athymic mice. The implantation of testosterone pellet further stimulated the growth of the tumor and a cell line was established from the tumor and named ZR-75-1/Aro-Clone10-TT1 (TT1). To detect their potential for early metastasis, we stably transfected a lentiviral EGFP and luciferase expression vector (a gift from Dr. Brian Rabinovich at MD Anderson Cancer Center) into 293T packaging cells. Forty-eight hours after the transfection we collected the conditioned medium and infected the ZR-75-1, Cl.10 and TT1 cells. After another 48 hour we changed the medium to regular medium and to confirm that the luciferase is functional, we checked the luciferase activity of these luciferase-transfected ZR-75-1, Cl.10 and TT1 cells. The human breast cancer cell line CAMA-1 cell line was a gift from Dr. Linda deGraffenried of UT Austin. MCF-7/Aro cell line was from Dr. Raj Tekmal at UTHSCSA. All these cell lines were cultured in McCoy's 5A medium supplemented with pyruvate, vitamins, amino acids, antibiotics, and 10% fetal bovine serum, as previously described[11]. Working cultures were maintained at 37°C in a humidified incubator with 5% CO_2_.

### Preparation of cell extracts and Western blot analysis

Cells were grown to confluence in 60-mm tissue culture dishes. For suspension culture, cells were grown in ultra low adherent tissue culture dishes. As per requirement of the experiment, the cells were grown for 24 h, 48 h or 96 h before harvesting. Cells were then lysed in buffer [50 mmol/L Tris-HCl (pH 7.4), 150 mmol/L NaCl, 0.5% NP40, and a protease inhibitor cocktail] and total protein concentration was obtained with the BCA protein assay (Pierce). Equal amounts of protein were separated with 10% or 7.5% SDS-PAGE and blotted onto nitrocellulose membranes. The blotted membranes were incubated with respective primary antibodies; mouse anti–glyceraldehyde-3-phosphate dehydrogenase (GAPDH; 1:10,000; Ambion, Inc.) was used as a loading control, horseradish peroxidase–conjugated goat anti-rabbit (1:5,000; Santa Cruz Biotechnology), and horseradish peroxidase–conjugated goat anti-mouse (1:5,000; Santa Cruz Biotechnology) antibodies were used as secondary antibodies.

### Antibodies

Antibody to the Estrogen Receptor alpha was from Labvision Corporation (Fremont, CA), and Santa Cruz Biotechnology (Santa Cruz, CA), antibody to Aromatase was from ABD Serotec (St. Louis, MO).

### Aromatase activity assay

To measure the aromatase enzymatic activity, the tritiated water release assay was used, using 3[H] androstenedione as the substrate. As described before[12], cells were grown in six-well cell culture plates, washed twice with PBS, and then incubated with 1 ml serum-free medium containing 500 nm progesterone and 100 nm [^3^H]androst-4-ene-3,17-dione as substrate. After 12-h incubation at 37°C, the reaction mixture was removed and extracted with an equal volume of chloroform to extract unused substrate and further treated with dextran-treated charcoal. After centrifugation, the ^3^H_2_O-containing supernatants were counted in a liquid scintillation counter. The protein concentrations were determined after dissolving cells in 0.5 m NaOH by the method of Bradford (Bio-Rad, Hercules, CA). Aromatase activity was calculated as femtomoles per milligram protein per hour.

### Apoptosis assay

Apoptosis was detected by using an apoptosis detection kit (Roche) after cells were treated as needed for 16 h. Cells were plated either in regular 6-well culture dishes for adherent culture or in ultra-low attachment 6-well plate for suspension culture. In suspension culture the cells were treated with 10 nM testosterone or 10nM testosterone + 1μM letrozole.

### Real time PCR assay

The mRNA levels were measured by real-time RT-PCR. Total RNA was isolated using TRIzol Reagent (Invitrogen) according to manufacturer’s instructions. RNA was reverse-transcribed using the M-MuLV RT from Promega. SYBR Green-based real time PCR assay was conducted following the manufacturer's procedures (Applied Biosystems for ABI7900). Actin was used for normalizing the real time PCR results. A specific forward primer was designed for measuring the mRNA level of exogenously transfected aromatase by targeting the prime to the 5’ end of the transfected exogenous aromatase transcript containing 5’ untranslated sequence from the vector. The sequence for the human aromatase primers are hArom-F 5′TGGAATTATGAGGGCACATCC3′ and hArom-R 5′GTCCAATTCCCATGCAGTAGC3′, which detected both endogenous and exogenous aromatase transcripts. Primers for exogeneous aromatase are EArom-F 5’AAATAGTCGGTGAAGAAACC3’ and EArom-R 5’CTTATCATGTCTGGATCCCT3’. Estrogen receptor-α primers are ER-α-F 5’CCACCAACCAGTGCACCATT3’ and ER-α-R 5’GGTCTTTTCGTATCCCACCTTTC3’.

### Tumorigenicity and *in vivo* metastasis study

Four- to five-week-old female athymic nude mice (obtained from Harlan Sprague Dawley, Inc., Indianapolis, IN) were used for *in vivo* animal experiments. The animals were housed under specific pathogen-free conditions. Luc-GFP expressing Cl.10 and TT1 cells were implanted orthotopically into the inguinal mammary fat pad area (2x10^6^ cells/side/mouse) of 5-week-old female athymic nude mice. Notably, both cell lines generated orthotopic tumors with no estrogen supplementation after three weeks post inoculation. The tumor sizes were measured with a caliper in two dimensions. Tumor volumes were calculated with the equation V = (L x W^2^) x 0.5, where L is length and W is width of a tumor.

### Experimental *in vivo* bone metastasis study

An intracardiac injection model for experimental bone metastasis was used for this study, as previously described [13]. Briefly, ZR-75-1 and Cl.10 cells were harvested from sub confluent exponentially growing cultures. The cells were injected into the left cardiac ventricle of anesthetized female nude mice (5 weeks old) with a 27-gauge needle attached to a 1-ml syringe using a micromanipulator. Each mouse was injected with 1 x 10^5^ cells in 0.1 ml of phosphate-buffered saline, and successful injections were indicated by the pumping of red blood into the syringe. Development of bone metastasis induced by Luc-EGFP-expressing cells was monitored at regular intervals by whole mouse fluorescence and bioluminescence imaging using Xenogen IVIS Spectrum System (Caliper Bioscience, CA) to detect Luc-EGFP-expressing tumor cells growing in the legs, arms, spine, and mandible bones. X-ray radiographs taken with Faxitron were used for the detection of any bone lesion in mice injected with these cells.

### Statistical Analysis

Two-tailed student t-tests or one way ANOVA were performed to determine the significant difference between control and experimental data. All the statistical analysis was performed with Graph Pad Prism 3.03 software.

## Results

### Human ERα+ breast cancer cell lines express detectable levels of aromatase transcript and aromatase protein

**Fig 1:**
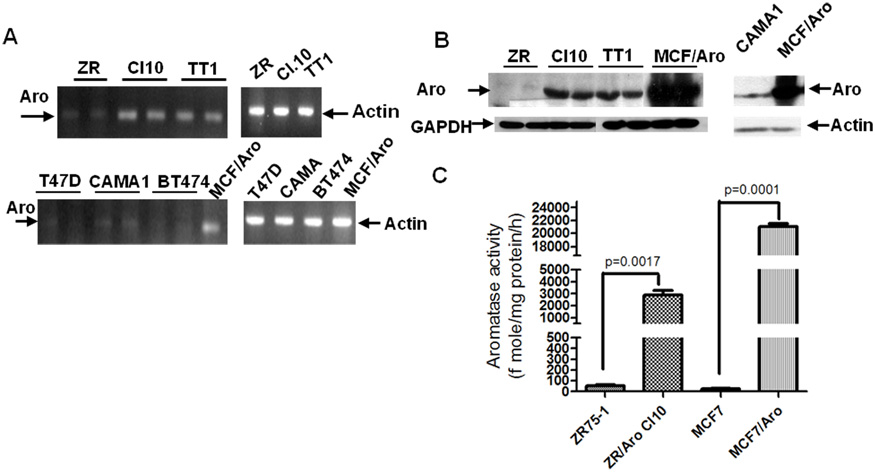
Aromatase expression levels in ERα+ breast cancer cell lines. **A.** mRNA levels of aromatase detected with RT-PCR in ERα+ breast cancer cell lines (ZR-75-1, ZR-75-1/Aro Cl.10, ZR-75-1/Aro-Clone10-TT1, T47D, CAMA-1 BT474). **ZR**: ZR-75-1 control cells stably transfected with an empty vector; **Cl.10**: a clone of ZR-75-1 cells stably transfected with an aromatase expression vector; **TT1**: cell line derived from a xenograft tumor formed by ZR-75-1/Aro Cl.10 cells in female nude mouse. **B.** Western immunoblotting of aromatase in various ERα+ cell lines. **C.** Aromatase enzyme activity in the extracts of ZR-75-1, ZR-75-1/Aro Cl.10, MCF-7 and MCF-7/Aro cell lines.

We found that human ERα+ breast cancer cell lines (ZR-75-1, CAMA1, T47D and BT474) express variable, but detectable levels of aromatase transcripts by real time RT PCR (Fig. 1A), and ZR-75-1 and CAMA1 cell lines also express detectable aromatase protein levels with Western immunoblotting (Fig. 1B). On the other hand, aromatase-transfected ZR-75-1 cells, Cl.10 and TT1 (see Materials and Methods for their generation), expressed aromatase mRNA and protein at a higher level than the breast cancer cell lines, but lower than previously reported [14] in aromatase transfected MCF-7 cells. Consistent with the expression levels, the aromatase activity assay showed that aromatase-transfected ZR-75-1 cells (Cl.10) had an intermediate level of aromatase enzyme activity when compared to control cells and MCF-7/Aro cells (Fig. 1C).

### Aromatase expression was increased in suspension culture

**Fig 2:**
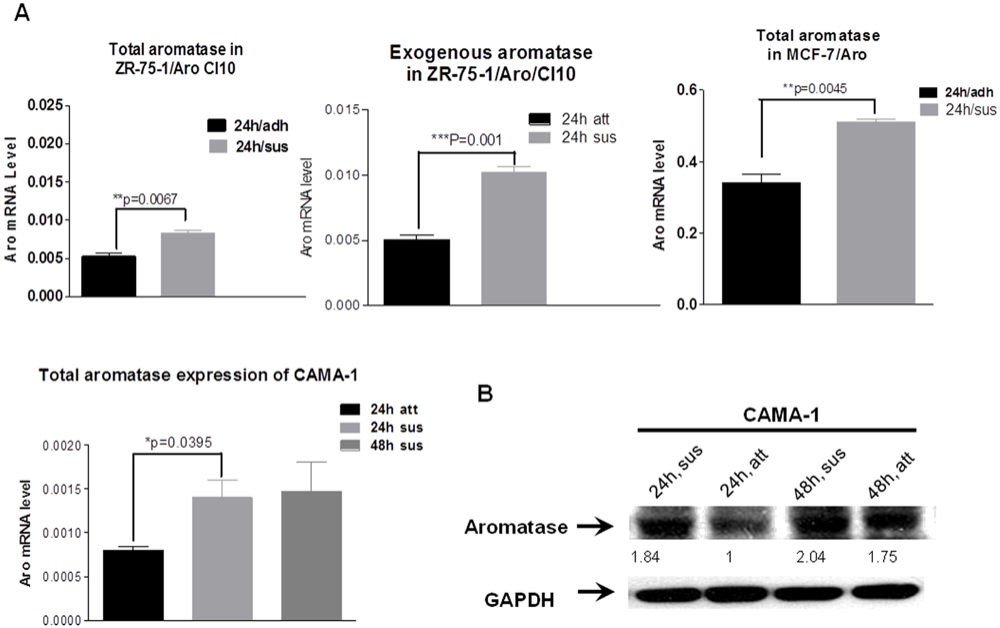
Increased expression of aromatase in suspension culture. Cells were plated at 1x10^6^ per well in regular plates for adhesion culture for 24 hrs (24h/adh) or in ultra-low adhesion plates for suspension culture for 24 hrs (24h/sus) and 48 hrs (48h/sus). Cells were harvested for RNA isolation, which was used in real time RT-PCR for quantification of aromatase and actin mRNA. Aromatase mRNA levels presented are normalized by actin mRNA levels. The data represents mean±SEM from three replicate measurements. Fig. A shows the increased aromatase expression in suspension culture of MCF-7, Clone 10 and CAMA-1 cells by real time PCR while Fig. B shows the same by Western blot analysis in CAMA-1 cells.

**Fig 3:**
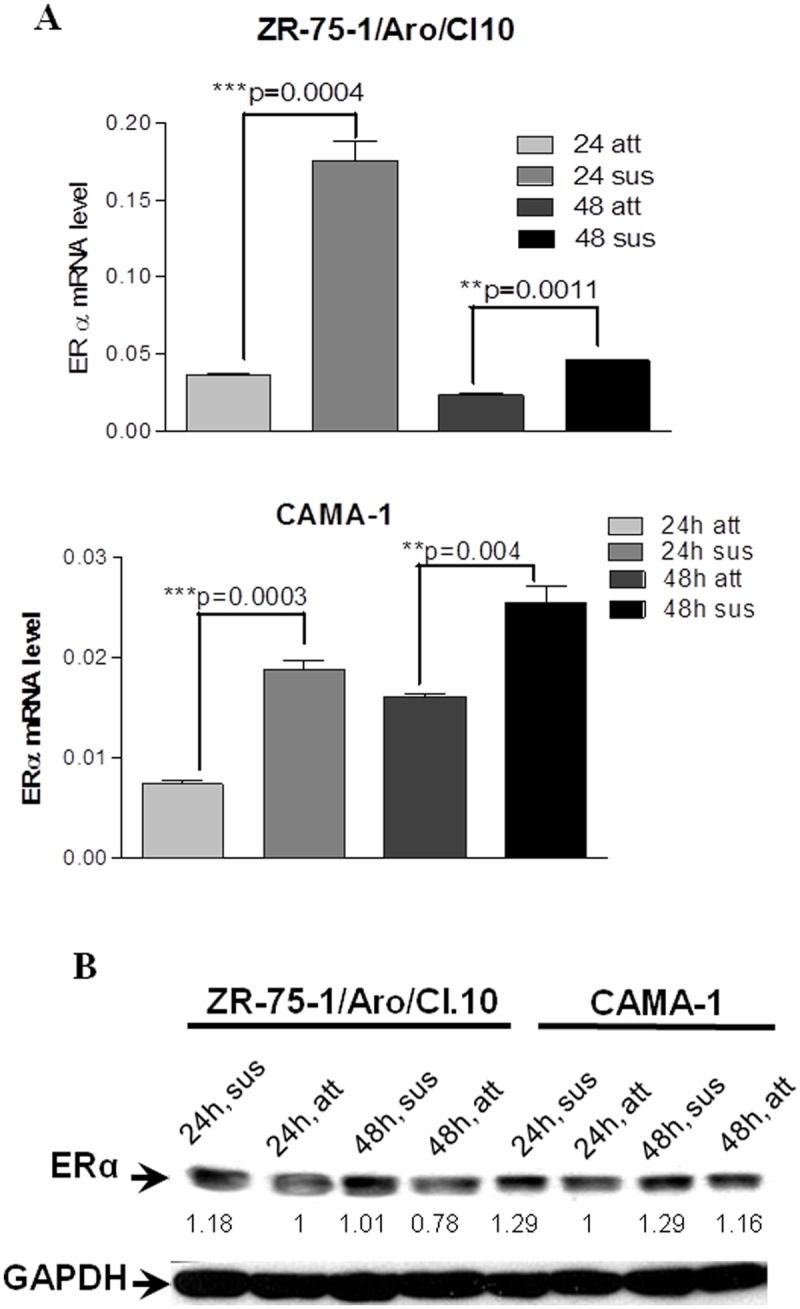
Increased expression of ERα in suspension culture. Cells were plated in either regular culture plates for adhesion culture or in ultra-low adhesion plates for suspension culture. Their RNA and protein were extracted and used for real-time RT-PCR (Panel A) or immunoblotting (Panel B). The ERα mRNA levels presented are normalized by actin mRNA. The data in Panel A represents mean±SEM from three replicate measurements. Two-tailed student t-tests were performed to determine the significant difference between control and experimental data. GAPDH protein was blotted to indicate equal loading in Panel B. The density of the ERα band from 24h adherent culture was set as one unit after being normalized with the corresponding GAPDH band for each cell line.

When CAMA-1 along with aromatase-transfected MCF-7(MCF7/Aro) and ZR75-1(ZR75-1/Aro/CL.10) cells were cultured in suspension in ultra-low adhesion culture plates to mimic the circulating tumor cells (CTCs), interestingly, all of these three ERα+ breast cancer cell lines expressed more aromatase in suspension culture when compared to the adhesion culture. Moreover the increase of aromatase expression has a positive correlation with the culture time in suspension condition and is statistically significant (Figs. 2A and 2B). We also demonstrated that both endogenous and exogenous (transfected) aromatase gene contribute to the upregulation of aromatase in suspension culture. Interestingly, the cells under suspension culture also showed increased ERα mRNA (Fig. 3A) and protein (Fig. 3B), which could increase the sensitivity of the breast cancer cells to intracrine estrogen.

### Increased aromatase and ERα expression led to anoikis resistance

**Fig 4:**
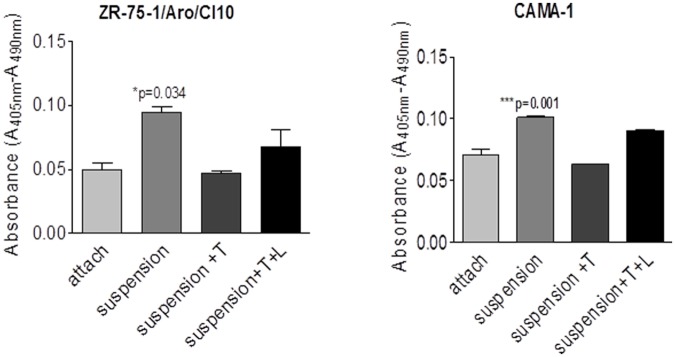
Aromatase-mediated inhibition of anoikis. Cells were plated as duplicate in ultra-low attachment 6-well plate for suspension culture. The cells were treated without or with 10 nM testosterone or 1 M letrozole, or both for 16 hr. Cell lysates were then used for apoptosis measurement with an apoptosis detection kit (Roche). Absorbance(A405nm-A490nm) indicates relative apoptosis. The data are presented as mean±SEM of duplicate wells (*p<0.05). One way ANOVA were used to find the significant difference.

Although transformed cells are resistant to suspension-induced programmed cell death called anoikis, many transformed cell lines still undergo significant apoptosis when suspended in culture. On the other hand, estrogen signaling is known to inhibit apoptosis. Thus, we investigated whether increased aromatase and ERα under suspension culture can inhibit anoikis. By performing the Cell Death Detection ELISA Assay, we found that suspension culture caused anoikis of ZR-75-1/Aro-Cl.10 and CAMA-1 cells (Fig. 4). But this suspension culture induced anoikis was shown to be inhibited by the supplementation of 10 nM testosterone, which is converted to estradiol by aromatase. Conversely, addition of the aromatase inhibitor letrozole blocked the testosterone-induced anoikis inhibition (Fig. 4) suggesting that the enhanced expression of aromatase and ERα in suspension culture likely increased estrogen synthesis and signalling [15–17].

### Aromatase expression rendered ERα+ breast cancer cells tumorigenic without estrogen supplementation

**Fig 5:**
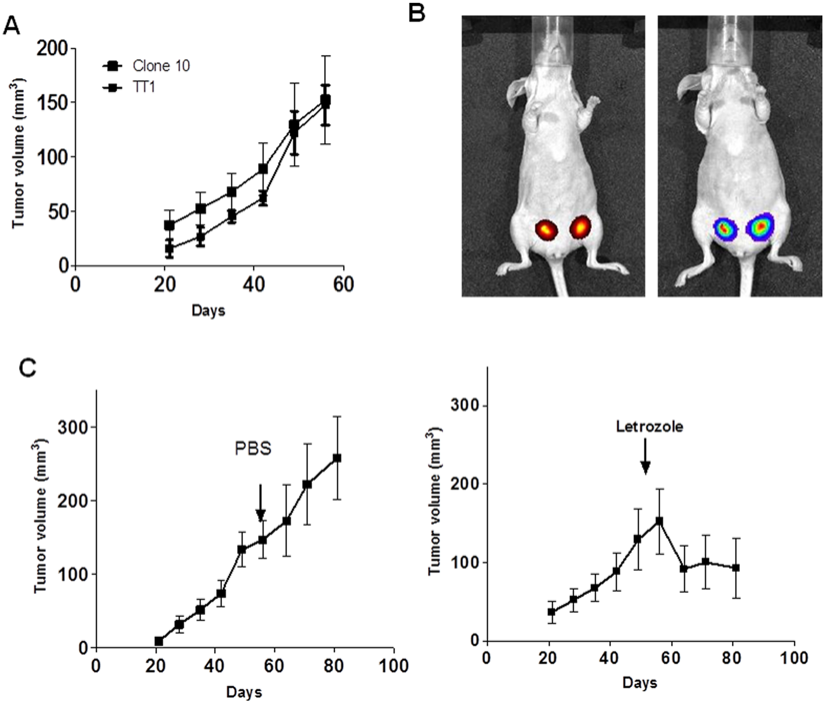
Moderate aromatase expression rendered ZR-75-1 cells tumorigenic without estrogen supplementation. **A.** Luc-GFP expressing ZR-75-1/Aro Cl.10 and TT1 cells (2x10^6^) were inoculated into the inguinal mammary fat pads of 5-wk-old female nude mice. The tumor sizes were measured with a caliper in two dimensions. Tumor volumes were calculated with the equation V = (LxW^2^)/2, where L is length and W is width of a tumor. Values are mean±SEM of 10 tumors in 5 mice. B. The representative images (fluorescence on the left and bioluminescence on the right) C. The mice with growing tumors were divided into two groups and treated with vehicle or letrozole at 10 μg/mouse/day.

To investigate the role of aromatase and potential intracrine estrogen signaling in promoting tumorigenesis, we implanted the Cl.10 and TT1 cells orthotopically into female athymic mice. The control ZR-75-1 cells were used as a control. Notably, a moderate expression level of ectopic aromatase caused Cl.10 and TT1 cells to become tumorigenic with no exogenous estrogen supplementation after three weeks of inoculation (Fig. 5A) whereas the control ZR-75-1 cells did not form any tumor. However, no significant differences in the tumor size were observed between Cl.10 group and TT1 group. Because the cells stably express both GFP and luciferase, the orthotopic tumors were also detected by fluorescence and bioluminescence imaging (Fig. 5B).

To determine whether growth of the orthotopic tumors was due to the ectopic aromatase expression and estrogen synthesis, we divided the mice bearing orthotopic tumors into two groups when the average tumor volume reached 150 mm^3^. One group was treated with the vehicle whereas the other group was treated with letrozole at 10 μg/mouse/day. Interestingly, the tumors started to shrink after letrozole treatment whereas the tumors in the vehicle group continued to grow (Fig. 5C). These results show that aromatase expression in ERα+ breast cancer cells can promote their malignancy.

### Aromatase expression maintained survival of ERα+ breast cancer cells in circulation and caused distant metastases

**Fig 6:**
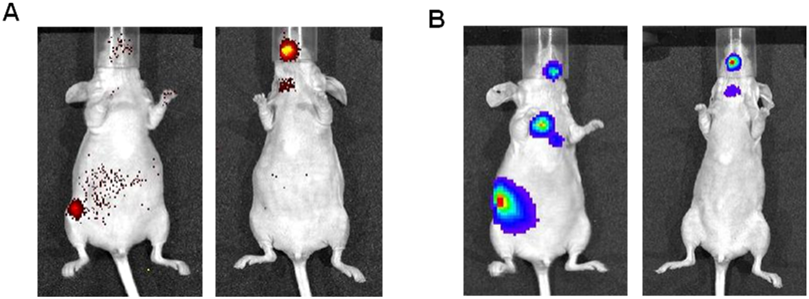
Aromatase expression rendered non-tumorigenic ZR-75-1 cells bone metastatic. The cells were inoculated through the left cardiac ventricle of female nude mice at 0.1x10^6^ cells/mouse. Bone metastases of ZR-75-1/Aro Cl.10 cells in the mandible and tibiae/femora were detected with fluorescence (panel A) and bioluminescence (panel B) imaging. Representative images of two mice inoculated with the Cl.10 cells are presented, which were taken 5 week post-inoculation.

To extend our study further in determining the importance of intracrine estrogen in promoting distant metastasis, we inoculated the Cl.10 cells and its control ZR-75-1 cells intracardiacally into the left ventricle of 5-wk-old female nude mice (10 mice in each group) and monitored metastatic tumor growth at regular interval by fluorescence and bioluminescence imaging. Interestingly it was observed that mice with intracardiacally inoculated Cl.10 cells presented distant bone metastasis in the mandible (80% incidence) and tibiae/femora (40% incidence) after 2-weeks of inoculation without estrogen supplementation (Fig. 6), while no metastasis was detected in the mice inoculated with the control ZR-75-1 cells.

## Discussion

Tissues in breast cancer have been demonstrated to express more aromatase than the normal breast tissues, which consequently lead to much higher level of estrogen in the local site. This is one of the main reasons that aromatase has drawn so much interest in the treatment of breast cancer [18]. An earlier study by Angela Brodie’s group[14] had shown that ectopic expression of Aro in the breast carcinoma of MCF-7 cells were more tumorigenic in nude mice when supplemented with an androgenic precursor androstenedione. Consistent with this study, our result showed the formation of subcutaneous tumors in the mice inoculated with TT1 cells without exogenous supplementation of estrogen, which indicate that moderate expression level of ectopic aromatase could increase the malignancy of ER-α (+) breast cancer cells. The local Aro expression has been shown to play a significant role in the development and progression of ERα+ breast cancer, hence aromatase inhibitors (AIs) are becoming preferred drugs to tamoxifen as they were found to be more effective than tamoxifen in blocking the progression of metastatic ERα+ breast cancer in postmenopausal patients[9]. In our study, the reduced size of the tumors and the blockage of tumor progression caused by application of letrozole at a dose of 10 μg/mouse/day confirmed that the tumorigenic feature of TT1 was mostly associated with ectopic aromatase expression.

Another recent study has shown that suspension culture can stimulate aromatase expression in adipose stromal cells[19]. In our study with stable clones from the human aromatase expression vector transfected ER positive (ER+) breast cancer ZR-75-1 cells and other ER-α (+) BCa, we show that ER-α (+) breast cancer cells in suspension culture express more aromatase. The increase in aromatase expression in suspension culture suggests that circulating ER-α (+) breast cancer cells may up-regulate intracrine estrogen activity for survival, after leaving the estrogen-rich adipose stroma at the primary site. Additionally, the high expression of ER-α was also observed in suspension cultured breast cancer cells, which could provide one potential explanation for the survival of ER-α positive breast cancer cells in circulation system. Interestingly, it was reported that when SK-BR-3 cells (an ER negative breast cancer cell line) were transfected with ER-α, estrogen induced the expression of aromatase[20]. mRNA levels of aromatase and ER-α were shown to have a moderate correlation in a clinical studies[21]. Thus, further studies are needed to determine whether the suspension culture induced expression of ER-α might upregulate the aromatase expression in breast cancer cells.

It is generally accepted that ER-α pathway acts as a breast cancer promoter by its induction of proliferation[22] and crosstalk with other signaling pathway[23]. However, couple of works has also demonstrated a role for estrogen on the suppression of apoptosis. A form of cell death due to loss of contact with the extracellular matrix or neighboring cells defined as anoikis is an important barrier for cancer cell metastasis. Although various pathway are implicated in the regulation of anoikis, ER-α signaling is believed to be a major modulator in the ER-α positive breast cancer acquiring anoikis resistance[24]. Consistent with the previous studies, our data suggests that the E2 produced by Aromatase reverse the apoptosis induced by the suspension culture. This result proved our hypothesis that the up-regulated autocrine aromatase support the survival of breast cancer cells in suspension condition.

Metastases are believed to be the major cause of death in breast cancer patients. It was reported that over 80% of breast cancer patients will develop skeletal spread before their demise[25], and approximately 40,000 women with breast cancer die from bone metastases each year[26]. For many years, tamoxifen has been considered as the standard endocrine therapy for postmenopausal women with hormone-sensitive breast cancer. However the BIG1-98 study showed that letrozole could reduce the risk of distant metastases by 27% in the HR+ population in the early period after diagnosis when compared with tamoxifen[26]. Another studies with breast cancer tissue samples also showed that high aromatase mRNA levels were significantly associated with disease progression and poor survival outcome[21]. Consistent with the clinical trial described above, our in vivo study indicated that ectopic expression of aromatase promoted the survival and distant bone metastasis of ERα+ breast cancer cells. The survival of ERα positive breast cancer cells in circulation and the formation of distant metastases are likely due to the excess production of estrogen that catalyzed by aromatase.

## Conclusions

Our studies show that a moderate expression of aromatase in the breast cancer cells is sufficient to generate intracrine estrogenic action. Aromatase expression promotes tumorigenesis and skeletal metastasis in the ERα positive breast cancer cells. These findings provide an important foundation for future investigation on how hormone-dependent breast cancer cells survive in circulation and metastatic sites during the process of metastasis. Our aromatase-expressing models should be useful for exploring how up-regulated aromatase expression contributes to the malignancy of ERα positive breast cancer cells.
